# An explainable machine learning-based clinical decision support system for prediction of gestational diabetes mellitus

**DOI:** 10.1038/s41598-022-05112-2

**Published:** 2022-01-21

**Authors:** Yuhan Du, Anthony R. Rafferty, Fionnuala M. McAuliffe, Lan Wei, Catherine Mooney

**Affiliations:** 1 UCD School of Computer Science, University College Dublin, Dublin, Ireland; 2 UCD Perinatal Research Centre, School of Medicine, University College Dublin, National Maternity Hospital, Dublin, Ireland

**Keywords:** Gestational diabetes, Computer science

## Abstract

Gestational Diabetes Mellitus (GDM), a common pregnancy complication associated with many maternal and neonatal consequences, is increased in mothers with overweight and obesity. Interventions initiated early in pregnancy can reduce the rate of GDM in these women, however, untargeted interventions can be costly and time-consuming. We have developed an explainable machine learning-based clinical decision support system (CDSS) to identify at-risk women in need of targeted pregnancy intervention. Maternal characteristics and blood biomarkers at baseline from the PEARS study were used. After appropriate data preparation, synthetic minority oversampling technique and feature selection, five machine learning algorithms were applied with five-fold cross-validated grid search optimising the balanced accuracy. Our models were explained with Shapley additive explanations to increase the trustworthiness and acceptability of the system. We developed multiple models for different use cases: theoretical (AUC-PR 0.485, AUC-ROC 0.792), GDM screening during a normal antenatal visit (AUC-PR 0.208, AUC-ROC 0.659), and remote GDM risk assessment (AUC-PR 0.199, AUC-ROC 0.656). Our models have been implemented as a web server that is publicly available for academic use. Our explainable CDSS demonstrates the potential to assist clinicians in screening at risk patients who may benefit from early pregnancy GDM prevention strategies.

## Introduction

Gestational Diabetes Mellitus (GDM) is generally defined as “glucose intolerance of varying degrees of severity with onset or first recognition during pregnancy”^1^. The risk of GDM is increased with overweight and obesity^2^, of which the global prevalence has increased substantially in the past decades^3^. GDM increases the risk of many maternal and neonatal complications such as gestational hypertension, polyhydramnios, Caesarean birth, premature delivery, large for gestational age, and neonatal macrosomia, intensive care unit admission, hypoglycaemia and respiratory distress^4^. Moreover, GDM may predispose to long-term sequelae for both mother and child including metabolic syndrome and type 2 diabetes mellitus^5^, thus increasing later life chronic disease.

Research shows that interventions initiated early in pregnancy can reduce the rate of GDM in pregnant women with overweight and obesity^6–8^. However, applying interventions in every instance can be costly and time-consuming. A clinical decision support system (CDSS) based on machine learning can be helpful in providing a powerful and objective computerised tool to assist clinicians identify women at risk of GDM. It would largely reduce the time and cost by allowing targeted intervention. The CDSS has great potential in clinical settings, especially under the circumstance that many clinicians have turned to telemedicine to maintain social distancing during the COVID-19 pandemic^9^.

CDSSs have a great potential to improve healthcare delivery, though literature on their successful adoption, especially that of machine learning-based CDSSs, is scarce. Shortliffe and Sepúlveda^10^ indicated that aside from system accuracy, efficiency and usability are important for a CDSS to be accepted and integrated into clinical workflow. A CDSS should be time-saving, intuitive and simple to use in order to obtain system outputs easily while juggling a heavy clinical workload. They also pointed out that black-boxes are not acceptable for CDSSs. This is inline with Antoniadi et al.^11^ who indicated that explainability is a critical component for a CDSS to be adopted in practical use effectively. A famous example by Caruana et al.^12^ shows that a machine learning-based system can reflect the pattern in the training data but be inconsistent with medical knowledge and thus does not translate to clinical practice. Their system predicted that patients who had a history of asthma had a lower risk of dying from pneumonia than the general population. This is because patients who had asthma and presented with pneumonia usually receive aggressive care which lowers their risk. Even though the system truly captured the training data, it would be problematic if adopted in clinical practice without understanding why the model behaved this way. Such problems can be resolved using Explainable Artificial Intelligence (XAI). Many benefits have been reported in the use of XAI in CDSS, including enhancing decision confidence, generating the hypothesis about causality, and increasing acceptability and trustworthiness of the system. Nevertheless, there is an overall distinct lack of the application of XAI in CDSSs in the published literature^11^.

We aim to apply machine learning to develop a CDSS that predicts the risk of GDM in a high risk group of women with overweight and obesity to identify those who may benefit from prevention strategies early in pregnancy. We performed modeling on baseline maternal characteristics and blood biomarkers collected in the Pregnancy Exercise and Nutrition with smart phone application support (PEARS) study. Multiple probabilistic prediction models incorporating different feature subsets were developed for different use cases, including theoretical, routine antenatal and remote risk assessment. Intelligent optimization algorithms were not considered in this work^13–16^. Clinical usability was taken into account throughout the modeling process. Moreover, we applied Shapley additive explanations (SHAP), a game theoretic XAI method, to explain the models and thus make them more acceptable and trustworthy for clinicians. The models were implemented as a web server-based CDSS that is open for academic use. Our CDSS has a potential to help clinicians identify women at risk of GDM in early pregnancy.

The rest of the paper is organised as follows. Section “Related work” reviewed previous research done in the related field. The data, modeling process and explanation method used in this research are introduced in Section “Methods”. Section “Results” describes our final models and their performance on white and non-white populations, as well as the implementation of a CDSS prototype and a case study. The discussion of our findings are presented in Section “Discussion”. Section “Conclusion” concludes this paper.

## Related work

We comprehensively reviewed original research articles published between 1 Jan 2015 and 1 Dec 2021 on the use of machine learning to predict the risk of GDM. The search was performed on PubMed, Science Direct, Scopus, IEEE and ACM. The search terms are: “machine learning”, “gestational diabetes mellitus” and “risk prediction”. A total of nine papers were deemed relevant and summarized in Table 1.

**Table 1:** Previously published machine learning-based GDM risk prediction models.

Authors	Subjects/data	Algorithm	Specificity	Sensitivity	AUC-ROC
Qiu et al.^17^	4,378 women	Cost-sensitive hybrid model of logistic regression, support vector machine and CHAID tree	0.998	0.622	0.847
Zheng et al.^18^	4,771 women	Multivariate Bayesian logistic regression	0.75	0.66	0.766
Ye et al.^19^	22,242 pregnancies	Gradient boosting decision tree	0.99	0.15	0.74
			0.26	0.90	
Artzi et al.^20^	588,622 pregnancies	Gradient boosting	–	–	0.854
Xiong et al.^21^	490 women	Light gradient boosting machine	0.995	0.883	0.942
Yan et al.^22^	3,988 women	Logistic regression	–	0.706	0.779
Hou et al.^23^	1,000 samples	Light gradient boosting machine	–	–	0.852
Wu et al.^24^	32,190 women	Deep neural network	0.82	0.63	0.80
Wu et al.^25^	17,005 women	Random forest	0.269	0.91	0.746
			0.524	0.75	
			0.777	0.487	

Where multiple models were developed, the best performing model is described.

Given the prevalence of GDM, there are usually many more non-GDM cases than GDM cases leading to unbalanced datasets. Some of the studies have not successfully addressed this class imbalance problem which may lead to the development of models that perform well for the majority class (non-GDM) only, i.e. high specificity but low sensitivity^17,24^. In addition, most models have not been designed for use in a clinical setting. All models focused on GDM prediction in general pregnant women, whereas prediction in a high-risk group would allow for more cost-effective GDM screening which would be more suitable for clinical use. Only Artzi et al.^20^ and Wu et al.^24^ considered the impact of the number or accessibility of features included on the clinical usability of the models in the modeling process. Additionally, Artzi et al.^20^ is the only group of researchers who included the explainability in the model design. However, they did not provide justification of why their model made a specific prediction for an instance (local interpretation). Moreover, due to the geographic area where maternal data were collected, all models are trained on data from women who are predominantly from the same ethnic group. There is a lack of studies that investigate the differences that might be associated with different cultural or ethnic backgrounds in GDM prediction. Additionally, there is a lack of implementation of the models into an open-access web server or application which allows for benchmarking or validation. There is a randomised controlled trial designed to screen for high-risk women using the model by Wu et al.^24^ and assess the effect of an individualised nutritional intervention to prevent GDM in this population^26^, but the model is not publicly available for academic use.

## Methods

### Study design and data

This research is a secondary analysis of the Pregnancy Exercise and Nutrition with smart phone application support (PEARS) study (ISRCTN29316280), a randomised controlled trial carried out at the National Maternity Hospital (NMH), Dublin, Ireland between 2013 and 2016^27^. The study was approved by ethical committee of National Maternity Hospital. All methods were carried out in accordance with relevant guidelines and regulations.

The PEARS study recruited 565 women with singleton pregnancy and a body mass index (BMI) 25-39.9 kg/m$^2$ at 10-15 weeks gestation to investigate the prevention of GDM using a behavioural antenatal lifestyle intervention supported by a smartphone application in pregnancies complicated by overweight and obesity. Maternal written informed consent was acquired at study enrolment. The PEARS participants were stratified by BMI and randomly assigned into control (standard antenatal care) and intervention (additional dietary and physical activity advice with reinforcement using mHealth technology) groups. During the PEARS study, a variety of data were collected from these participants, including maternal characteristics, blood biomarkers, ultrasound measurements, food intake, exercise and lifestyle behaviors, maternal and neonatal outcomes. GDM was diagnosed according to the International Association of the Diabetes and Pregnancy Study Groups (IADPSG) criteria^28^ at approximately 28 weeks gestation.

In this research, we excluded PEARS participants who dropped out or were excluded from the PEARS study, and those without available GDM diagnostics. As a result, 484 PEARS participants were included. Because the PEARS study did not find any significant difference between the incidence of GDM in the control and intervention group, we did not consider the impact of the PEARS intervention in this research.

As we aimed to predict GDM in early pregnancy, the descriptive features used are clinical data collected at the PEARS randomisation visit (14.91±1.66 weeks gestation, also referred to as the baseline). These features are maternal anthropometry, demographic characteristics, family history and blood biomarkers. They are described in Table 2.

**Table 2:** Descriptive features for gestational diabetes mellitus prediction.

Feature	Non-GDM (n=413)	GDM (n=71)
*Numerical*	*Mean (SD)*	*Mean (SD)*
Gestational Age (Weeks)	14.94 (1.66)	14.76 (1.62)
Maternal Age (Years)	32.35 (4.44)	33.80 (3.98)
Pobal HP Deprivation Index	6.07 (11.30)	7.11 (10.83)
Parity	0.71 (0.90)	0.91 (1.02)
Height (m)	1.64 (0.07)	1.64 (0.07)
Weight (kg)	78.71 (10.72)	81.72 (10.86)
Body Mass Index (BMI) (kg/m$^2$)	29.06 (3.21)	30.23 (3.67)
Mid Upper Arm Circumstance (MUAC) (cm)	30.90 (2.47)	31.33 (2.47)
White Cell Count ($10^9$/L)	8.64 (1.89)	9.28 (1.72)
Fasting Glucose (mmol/L)	4.50 (0.29)	4.87 (0.38)
Insulin (mU/L)	8.99 (4.03)	11.11 (5.36)
C-peptide (ng/mL)	1.38 (0.58)	1.79 (0.93)
Total Cholesterol (mmol/L)	5.45 (0.94)	5.20 (0.88)
High-Density Lipoprotein (HDL) Cholesterol (mmol/L)	1.52 (0.43)	1.48 (0.45)
Low-Density Lipoprotein (LDL) Cholesterol (mmol/L)	3.30 (0.91)	3.06 (0.88)
Triglycerides (mmol/L)	1.39 (0.49)	1.45 (0.48)
Complement Component 3 (C3) (mg/dL)	156.04 (26.02)	166.88 (28.16)
C-Reactive Protein (CRP) (mg/L)	3.06 (7.12)	6.11 (16.22)
Leptin (ng/mL)	40.98 (19.36)	46.71 (24.72)
Adiponectin ($\mu$g/mL)	17.55 (9.87)	12.39 (5.67)
*Categorical*	*Number (%)*	*Number (%)*
Ethnicity		
White Irish	312 (75.54)	49 (69.01)
Other White	66 (15.98)	12 (16.90)
Black	5 (1.21)	0 (0)
Chinese	5 (1.21)	2 (2.82)
Other Asian	11 (2.66)	4 (5.63)
Mixed	5 (1.21)	2 (2.82)
Not Specified	9 (2.18)	2 (2.82)
Education		
Level 1: No schooling	0 (0)	0 (0)
Level 2: Primary school education only	0 (0)	0 (0)
Level 3: Some secondary education only	10 (2.42)	1 (1.41)
Level 4: Complete secondary education only	48 (11.62)	7 (9.86)
Level 5: Some third degree education only	84 (20.34)	17 (23.94)
Level 6: Complete third degree education	262 (63.44)	43 (60.56)
Family History of Diabetes Mellitus (DM)		
1: Yes	78 (18.89)	26 (36.62)
2: No	328 (79.42)	45 (63.38)

**Figure 1:**
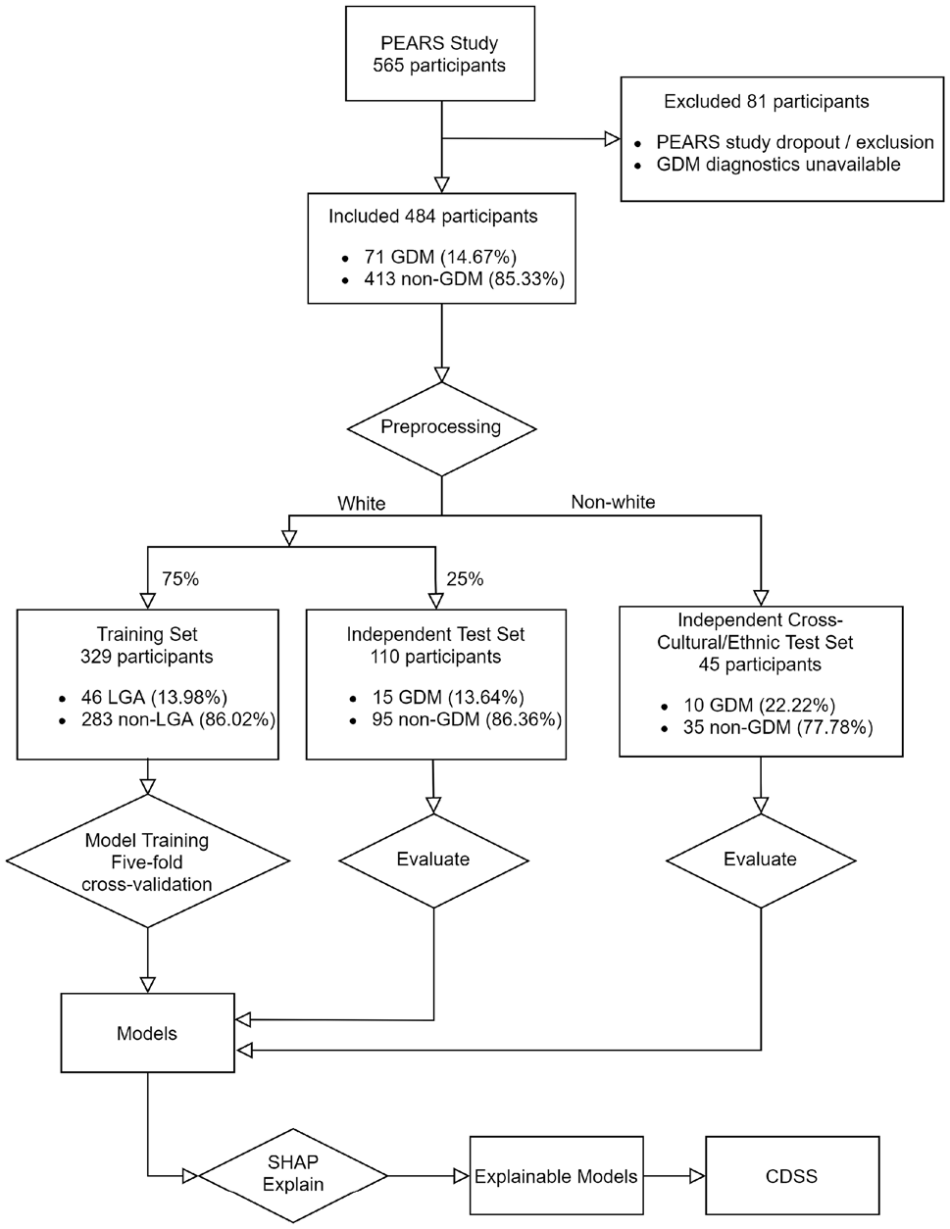
Workflow diagram.

Figure 1 shows the workflow overview of this research.

### Data preparation

**Figure 2:**
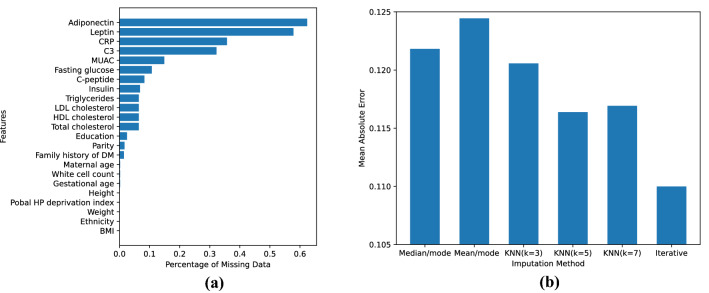
(**a**) Percentage of missing values for each feature. (**b**) Mean absolute error for generated missing values using different imputation methods.

Many features included in this research have missing values, as shown in Fig. 2a. We dropped features with greater than 30% missing values: complement component 3 (C3), C-reactive protein (CRP), leptin and adiponectin. The missing values in the remaining features were imputed. Several imputation methods were considered: median or mean for numerical features and mode for categorical features, k-Nearest Neighbors (kNN) imputation, and iterative imputation. In order to select the best imputation method for our dataset, we extracted the 313 complete cases of the dataset and generated missing values in these cases following the same missing pattern in the entire dataset. This is done by randomly selecting instances from the entire dataset and copying the feature missingness in these instances into the complete cases. Then we imputed these generated missing values using the candidate imputation methods and calculated the mean absolute error (MAE) between the imputed and true values after a min-max scaler. As shown in Fig. 2b, mean/mode imputation achieved the highest MAE, followed by median/mode imputation and kNN imputation with different parameters. Iterative imputation achieved the lowest MAE, showing that this method is able to generate imputed values closest to the real values for this dataset. Therefore, we applied the iterative imputation to impute the missing values in the dataset.

90.70% of the participants included were white, therefore, participants from other ethnic origins were held out as an independent cross-cultural/ethnic test set. After that, 75% of the white participants in the dataset, stratified by the GDM diagnosis, were randomly selected as the training set. The remaining 25% was used as an independent test set.

### Feature selection

**Figure 3:**
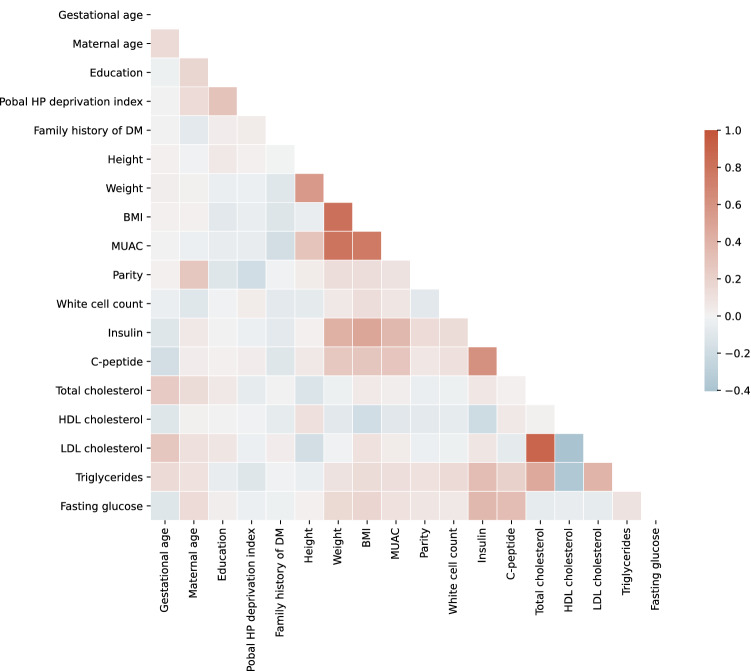
Correlation plot.

First of all, we aimed to remove redundant features to avoid the “curse of dimensionality”^29^ and reduce the number of inputs required to use the models in a clinical setting. Figure 3 shows the Pearson correlation between features. We defined redundant features as those with correlation greater than 0.6. As a result, maternal weight, BMI and mid-upper arm circumference (MUAC), insulin and C-peptide, and total cholesterol and Low-Density Lipoprotein (LDL) cholesterol were deemed to be redundant. This is consistent with the medical knowledge: weight, BMI and MUAC are all anthropometric measurements that provide assessment of nutritional status, and BMI is derived from height and weight. Insulin and C-peptide are released from the pancreas at the same time and the same rate. LDL cholesterol is calculated from total cholesterol, High-Density Lipoprotein (HDL) cholesterol and triglycerides. To reduce feature redundancy, we removed features with higher percentage of missing values prior to imputation (see Fig. 2a) to minimise the impact of imputation and ensure the credibility of data. As a result, MUAC and C-peptide are removed. The remaining redundant features that have the same level of missing values are: weight and BMI, and LDL cholesterol and total cholesterol. The latter of each pair is calculated from the former. Among them, we removed derived features (BMI, LDL cholesterol) and kept the original features (weight, total cholesterol) to save computational time.

Moreover, we conducted the feature selection process in close collaboration with clinical experts. In the existing literature, there is conflicting evidence reporting both positive and negative relationships with GDM and maternal education level, a indicator of socioeconomic status^30^. In our dataset, the average education level is higher in GDM participants than non-GDM participants, which can be explained by the positive relationship between maternal age and education. This is consistent with the indirect effect of maternal education on GDM through high pre-pregnancy BMI and older age that Bertolotto et al.^31^ suggested based on data collected in Italy. However, a cohort study in the Netherlands suggests low-educated women have an increased risk of GDM^32^. In order for our modeling results to be generalisable among different populations, and to avoid confusion in a clinical setting, we removed education in consultation with clinical experts.

In order to develop a clinically usable CDSS, we aimed to develop multiple models for different use cases. Model 1 was designed to be theoretical and feature-agnostic, where all included features were considered candidates and acts as a baseline. Model 2 was designed to be more usable and to fit into clinical routine easily. Fasting blood biomarkers (fasting glucose, insulin, C-peptide, lipid profile (total cholesterol, HDL cholesterol, triglycerides)) were excluded, because pregnant women do not normally attend an antenatal visit fasted and fasting blood biomarkers are not routinely assessed. Also, excluding these features can reduce the cost for a GDM prediction. Pobal HP deprivation index, a measurement of the women’s socioeconomic status based on the geographical area of their residence, was excluded because it is only available in Ireland and it limits the applicability of our model to other regions. Model 3 was designed to work in remote settings without a hospital visit, so all features that cannot be recalled or measured outside of a clinical setting were excluded. In addition to features removed in Model 2, for example, all blood biomarkers. Also, Pobal HP deprivation index was excluded. All included features for each model are further selected during cross-validation (described in Section “Modeling”).

### Modeling

We adopted several machine learning algorithms, namely logistic regression, random forest, support vector machine (SVM), adaptive boosting (AdaBoost) and extreme gradient boosting (XGBoost) for data modeling. In our dataset, 13.90% (61) of white women were diagnosed with GDM at approximately 28 gestational weeks, which means the dataset is highly unbalanced. For each model, we applied sequential steps of synthetic minority oversampling technique (SMOTE), feature selection by the highest ANOVA F-values and machine learning with each algorithm on the training set in a pipeline. The number of nearest neighbors used to construct synthetical samples in SMOTE is set to three. The number of top features to select (from one to the total number of candidate features) as well as hyper-parameters for each algorithm were tuned, using balanced accuracy as the evaluation metric, in a stratified five-fold cross-validated grid search. The hyper-parameters tuned for each algorithm can be found in Table 3. Probability estimates are enabled so that the models’ false positive rate are adjustable for different settings. All other parameters were set to the default.

In this research, scikit-learn 0.24.2^33^, imbalanced-learn 0.8.0^34^, fancyimpute 0.5.5^35^, xgboost 1.3.1^36^ and shap 0.39.0^37^ libraries were used for data processing. The developed algorithm was implemented in Python 3.8.8 [MSC v.1916 64 bit (AMD64)] and IPython 7.22.0 in Jupyter notebook 6.3.0 from Anaconda. This method runs on a Windows 10 PC with Intel(R) Core(TM) i7-7500U CPU @ 2.70GHz and 8GB RAM.

**Table 3:** Hyper-parameters for each algorithm in the grid search.

Logistic regression	C: 0.1, 1, 10
	solver: newton-cg, lbfgs, liblinear, sag, saga
	penalty: l1 (liblinear, saga solver only), l2, elasticnet (saga solver only)
Random forest	n_estimators: 100, 200, 300, 500
	max_depth: 10, 20, 30, 50
	max_features: auto, sqrt
	min_samples_leaf: 1, 2, 4
	min_samples_split: 2, 5, 10
Support vector machine	kernel: rbf, poly, sigmoid, linear
	C: 0.1, 1, 10
	degree: 2, 3, 4 (poly kernel only)
	gamma: scale, auto (rbf, poly, sigmoid kernel only)
Adaptive boosting	n_estimators: 20, 50, 100
	learning_rate: 0.1, 0.2, 0.3
Extreme gradient boosting	n_estimators: 20, 50, 100
	learning_rate: 0.1, 0.2, 0.3
	max_depth: 4, 6, 8
	objective: binary:logistic
	subsample: 0.6, 0.8, 1
	colsample_bytree: 0.6, 0.8, 1

### Evaluation

The models were tested on the independent test set. AUC-PR (area under curve of precision versus recall) and AUC-ROC (area under curve of sensitivity versus false positive rate) were used to evaluate the overall performance of the probabilistic prediction model. The models’ specificity, sensitivity, and balanced accuracy (ACC) were evaluated at a decision threshold of 0.5. The equations for these evaluation metrics can be found in (1) - (5) .

1 $$\begin{aligned} Precision= & {} \frac{TP}{TP+FP} \end{aligned}$$

2 $$\begin{aligned} Recall/Sensitivity= & {} \frac{TP}{TP+FN} \end{aligned}$$

3 $$\begin{aligned} Specificity= & {} \frac{TN}{TN+FP} \end{aligned}$$

4 $$\begin{aligned} False\,Positive\,Rate= & {} \frac{FP}{TN+FP} \end{aligned}$$

5 $$\begin{aligned} Balanced\,ACC= & {} \frac{Specificity+Sensitivity}{2} \end{aligned}$$

where:

- True positives (TP): the number of GDM cases that are predicted GDM
- False positives (FP): the number of non-GDM cases that are predicted as GDM
- True negatives (TN): the number of non-GDM cases that are predicted as non-GDM
- False negatives (FN): the number of GDM cases that are predicted as non-GDM

In addition, to investigate the effect of the imputation, we compared the models’ performance on the entire independent test set with that on the complete cases in the independent test set.

Finally, the models’ performance on the independent cross-cultural/ethnic test set is evaluated using the same evaluation metrics to investigate if the models, which are trained on white data, generalise well to non-white population.

### Explainability

Logistic regression models are inherently transparent and explainable. Random forest, SVM, AdaBoost and XGBoost models are more algorithmic complex making it more difficult for clinicians to understand how predictions are made. For such models, we applied Kernel SHAP^37^, a model-agnostic game theoretic post-hoc interpretation method, to improve model explainability. SHAP is a unified approach of feature importance with desirable properties, and it provides both explanations for the structure of the model (global explainability) and for a specific prediction (local explainability). SHAP was applied to improve explainability for a machine learning-based CDSS in neurology^38^. In our research, global feature importance is calculated by the averaged absolute SHAP values of the entire independent test set for each feature to help users gain insight of the overall behavior of the model. Furthermore, we used SHAP values of a particular instance to explain how each feature and its value contribute to the predicted GDM risk for this participant.

## Results

The majority of the participants included were white (90.70%) and predominantly white Irish (74.59%). All participants had at least some secondary education and most women (83.88%) had at least some third degree education. At baseline, maternal age was 32.57±4.40 years, height 1.64±0.07 m, body weight 79.15±10.78 kg and BMI 29.23±3.30 kg/m$^2$. At approximately 28 weeks gestation, 71 (14.67%) were diagnosed with GDM and 413 (85.33%) were not.

### Feature and algorithm selection

**Figure 4:**
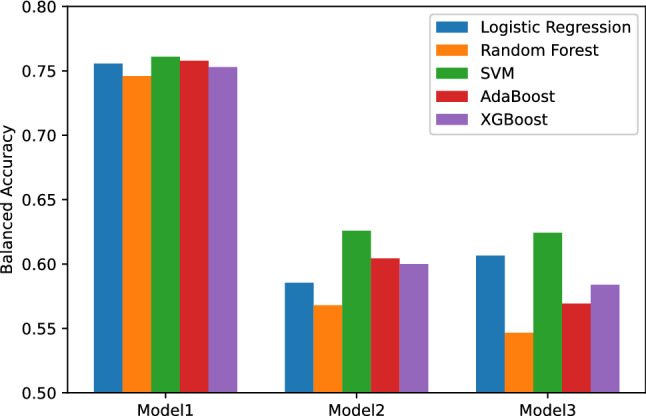
Models’ balanced accuracy in cross-validation.

To select the best machine learning algorithm for our models, we compared the balanced accuracy of different algorithms with optimal feature sizes and hyper-parameters evaluated in cross-validation. As shown in Fig. 4, SVM achieved the highest balanced accuracy for Model 1, 2 and 3 (0.761, 0.626, 0.624 respectively), and was thus selected over the other algorithms. For Model 1, five features are included in the optimal feature set of this model: family history of diabetes mellitus (DM), weight, white cell count, fasting glucose and insulin. For Model 2, five features are included in the model. They are: gestational age, maternal age, family history of DM, weight and white cell count. For Model 3, four features are included in the final feature set: gestational age, maternal age, family history of DM, and weight.

### Model evaluation

Table 4 shows the performance of models evaluated on the independent test set as well as the complete cases in the independent test set. Model 1 performs the best, achieving the AUC-PR of 0.485 and AUC-ROC of 0.792 on the independent test set. At the decision threshold of 0.5, the model predicts 73.3% of the white GDM participants and 76.8% of the white non-GDM participants correctly, giving a balanced accuracy of 75.1%. The performance of the model on the complete cases is slightly higher than on the independent test set. The model achieved the AUC-PR of 0.551, AUC-ROC of 0.860, and a balanced accuracy of 79.4% at decision threshold of 0.5.

Model 2 gives a lower but acceptable performance, with an AUC-PR of 0.208 and AUC-ROC of 0.659 on the independent test set. At the decision threshold of 0.5, Model 2 predicts 60% of the white GDM participants and 60% of the white non-GDM participants correctly, which gives a balanced accuracy of 60%. On the complete cases, the model achieved similar performance, giving an AUC-PR of 0.256, an AUC-ROC of 0.690, and a balanced accuracy of 59.2% at decision threshold of 0.5.

Model 3 performs similarly to Model 2. Model 3 achieved an AUC-PR of 0.199 and an AUC-ROC of 0.656 on the independent test set. At decision threshold of 0.5, 53.3% of the white GDM participants and 67.4% of the white non-GDM participants are predicted correctly, which gives a balanced accuracy of 60.4%. On the complete cases, the model achieved an AUC-PR of 0.320, an AUC-ROC of 0.687 and a balanced accuracy of 60.4%, which are similar to that on the independent test set.

Model 1 outperforms Model 2 and 3, which can be explained by the exclusion of fasting blood biomakers in Model 2 and 3. This indicates that fasting blood biomarkers, especially fasting glucose and insulin, are strong predictors of GDM. Our models perform similarly on the entire independent test set and the complete cases in the independent test set, showing that the imputation has little effect on model evaluation.

**Table 4:** Model performance on the entire independent test set and complete-case independent test set.

Model	Test cases	AUC-PR	AUC-ROC	Sensitivity	Specificity	Balanced accuracy
1	All (110 cases)	0.485	0.792	0.733	0.768	0.751
1	Complete (77 cases)	0.551	0.860	0.833	0.754	0.794
2	All (110 cases)	0.208	0.659	0.6	0.6	0.6
2	Complete (77 cases)	0.256	0.690	0.583	0.6	0.592
3	All (110 cases)	0.199	0.656	0.533	0.674	0.604
3	Complete (77 cases)	0.320	0.687	0.5	0.708	0.604

Model 1: feature-agnostic model. Features: family history of diabetes mellitus, weight, white cell count, fasting glucose, insulin.

Model 2: clinical routine model. Features: gestational age, maternal age, family history of diabetes mellitus, weight, white cell count.

Model 3: remotely usable model. Features: gestational age, maternal age, family history of diabetes mellitus, weight.

### Comparison between white and non-white populations

Table 5 shows the performance of models evaluated on the independent cross-cultural/ethnic test set compared with the independent test set. On the independent cross-cultural/ethnic test set, Model 1 achieved good overall performance, comparable to the performance on the independent test set. The model achieved the AUC-PR of 0.572 and AUC-ROC of 0.717. However, the model gives higher specificity than sensitivity at the decision threshold of 0.5. It predicts 60% of the non-white GDM participants and 80% of the non-white non-GDM participants correctly, giving a balanced accuracy of 70%.

Model 2 achieved an AUC-PR of 0.263 and an AUC-ROC of 0.643 on the independent cross-cultural/ethnic test set, comparable to that on the independent test set. However, at the decision threshold of 0.5, 68.6% of non-white non-GDM participants and only 30% of non-white GDM participants are correctly predicted.

Model 3 achieved similar overall performance on the non-white and white population, giving an AUC-PR of 0.293 and an AUC-ROC of 0.677. However, at the decision threshold of 0.5, the model is able to correctly predict 82.9% of non-white non-GDM participants and only 30% of non-white GDM participants.

**Table 5:** Model performance on the independent test set and independent cross-cultural/ethnic test set.

Model	Test population	AUC-PR	AUC-ROC	Sensitivity	Specificity	Balanced accuracy
1	Non-white (45 cases)	0.572	0.717	0.6	0.8	0.7
1	White (110 cases)	0.485	0.792	0.733	0.768	0.751
2	Non-white (45 cases)	0.263	0.643	0.3	0.686	0.493
2	White (110 cases)	0.208	0.659	0.6	0.6	0.6
3	Non-white (45 cases)	0.293	0.677	0.3	0.829	0.564
3	White (110 cases)	0.199	0.656	0.533	0.674	0.604

Model 1: feature-agnostic model. Features: family history of diabetes mellitus, weight, white cell count, fasting glucose, insulin.

Model 2: clinical routine model. Features: gestational age, maternal age, family history of diabetes mellitus, weight, white cell count.

Model 3: remotely usable model. Features: gestational age, maternal age, family history of diabetes mellitus, weight.

From the comparison of the model performance on the independent test set and the independent cross-cultural/ethnic test set, we found that our models, especially Model 2 and 3, achieved high specificity but low sensitivity at the decision threshold of 0.5 on the independent cross-cultural/ethnic test set, although similar overall performance (AUC-PR and AUC-ROC) was observed. In order to achieve an unbiased performance, a lower decision threshold for non-white population is required. As a result, we conclude that our GDM prediction models require a lower decision threshold to generalise to a non-white population.

### Implementation and worked example

Our models have been implemented as a web server, which serves as a CDSS prototype. The prototype allows users to submit the values for features required and it predicts the probability that the person will develop GDM. It also provides explanations in order for users to understand and trust the predictions. The prototype is freely available for academic use at http://lisda.ucd.ie/GDM-risk-calculator.

To test the prototype, we selected two test cases, which are similar but have different outcomes, from the independent test set (see Table 6). The two cases have similar values in most of the maternal characteristics except for weight: Case 1, the GDM participant, has extremely high weight, while Case 2, the non-GDM participant, has below average weight. Regarding blood biomarkers, Case 1 and 2 have similar white cell count, but Case 1 has higher fasting glucose and insulin than Case 2.

**Table 6:** Independent test cases.

Case	Outcome	Gestational age (weeks)	Maternal age (years)	Family history of DM	Weight (kg)	White cell count ($10^9$/L)	Fasting glucose (mmol/L)	Insulin (mU/L)
1	GDM	14	30.48	2: no	99.2	9.8	4.8	11.29
2	non-GDM	14	30.08	2: no	75.5	9.9	4.5	8.42

Suppose a user wants to test our CDSS prototype on these cases. They can simply enter the feature values for each case into the corresponding input boxes on the web page and press the “calculate” button to get the results. The CDSS will present the predicted risk for the case as well as the explanations.

Both cases were predicted correctly by Model 1, 2 and 3. The predicted GDM risks for Case 1 and 2 are 0.68 and 0.34 by Model 1, 0.68 and 0.44 by Model 2, and 0.54 and 0.42 by Model 3.

**Figure 5:**
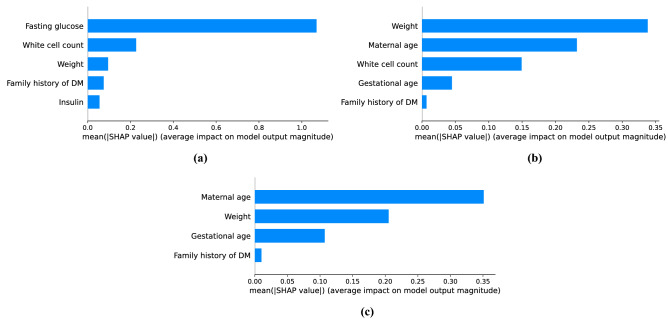
Global feature importance based on mean absolute SHAP value for (**a**) Model 1 (**b**) Model 2 (**c**) Model 3.

Our models are SVM-based models, and they are explained by Kernel SHAP in a post-hoc manner. Figure 5 shows the features ranked by their the global feature importance based on mean absolute SHAP values in all models. Fasting glucose plays the most important role in Model 1, achieving mean absolute SHAP values much higher than all other features. White cell count and weight are then of importance, followed by family history of DM and insulin. In Model 2, weight is the most important feature, followed by maternal age and white cell count. Gestational age and family history of DM play a less important role in Model 2. In Model 3, maternal age is the most important predictor, followed by weight, gestational age and family history of DM.

**Figure 6:**
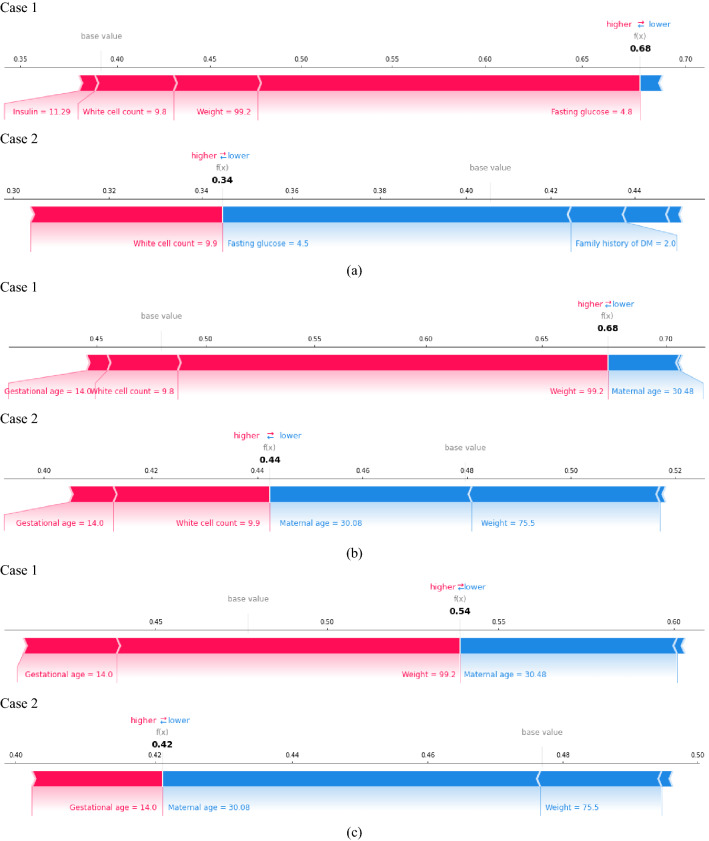
Local interpretation based on SHAP values for (**a**) Model 1 (**b**) Model 2 (**c**) Model 3.

Figure 6 shows the the local feature contribution based on SHAP values for Case 1 and 2 as well as the predicted GDM risk. A blue band indicates that the feature is dragging the prediction down to class non-GDM, while a pink band shows that the feature leads to an increase in the predicted GDM risk. The length of the band indicates the magnitude of the effect. It can be seen that the feature contributing the most to the opposite outcome of Case 1 and 2 in Model 1 is fasting glucose. The fasting glucose of 4.8 mmol/L largely increases the predicted GDM risk for Case 1, but the fasting glucose of 4.5 mmol/L decreases the risk for Case 2 greatly. This makes sense because Case 1 has fasting glucose close to the average in GDM participants but Case 2 has fasting glucose equal to the average in non-GDM participants (see Table 2). In Model 2 and 3, the features that contribute the most to the opposite outcome of Case 1 and 2 is weight. A weight of 99.2 kg largely increases the predicted GDM risk for Case 1, but a weight of 75.5 kg decreases the risk for Case 2. This makes sense because Case 1 has extremely high weight but the weight of Case 2 is lower than average.

In conclusion, the case study provides illustrative examples of the applicability of our CDSS prototype, and it shows that SHAP is able to reliably explain the individual predictions made by our models.

## Discussion

This is the first machine learning study that specifically targets pregnant women with overweight and obesity for GDM prediction. As opposed to previously published models (see Table 7) that predict GDM among all pregnant women, we focused on an at-risk group rather than prediction of GDM in a general pregnant population. Targeting a high-risk group is more helpful for clinicians as GDM is more difficult to identify in this cohort and it would allow GDM screening in an efficient manner in a clinical setting. Also, this can be particularly helpful in clinical practice if combined with a mobile health-supported lifestyle intervention that has been proven cost-effective in pregnant women with an elevated BMI^39^.

Our dataset is highly unbalanced as the non-GDM participants outnumber GDM participants greatly. This is also the case in previous works due to the prevalence of GDM. Unless properly handled, it can lead to a model that only performs well on the majority (non-GDM) class, i.e. achieves high specificity but low sensitivity (see^17,24^). We applied SMOTE to oversample the minority class in the training data. All of our models achieved similar specificity and sensitivity, showing that the class imbalance problem is adequately addressed. In addition, we used balanced accuracy instead of accuracy when testing, because the former gives a better evaluation of the model performance in a class imbalance setting. On the contrary, accuracy is likely to overestimate the overall performance of a model when it only performs well on the majority class.

An advantage of the study is that we carefully took the clinical usability into account in the modeling process. Three models were developed: Model 1 which included all features available including fasting blood biomarkers to assess GDM risk in a theoretical way; Model 2 which excluded fasting blood biomarkers for GDM prediction in a clinical routine; and Model 3 including self-reportable features only for remote use. In contrast to our work, most models in the literature included data from clinical tests that are not routinely performed in an Irish clinical setting (see Table 7), including fasting blood test as well as other tests that are not available in our dataset, such as coagulation function test and gene testing. The inclusion of these data may lead to an increase in the model performance, but at the cost of usability because it makes the models difficult to translate into clinical use. Our Model 1 performs well achieving AUC-ROC of 0.792. This is slightly lower than some published models which included fasting blood tests, because we included fewer blood tests and have focused on a high risk group of women with overweight and obesity among whom GDM prediction is more difficult compared to a general pregnant population. Our Model 2 and 3, although they gave lower performance than Model 1, have greater potential in a clinical setting as the features they use are easier to access clinically. Compared with previous works, our Model 2 included only one feature from routine blood tests, whereas published models included at least two blood test-related features. Our Model 3 is the first model that does not include any blood test. To the best of our knowledge, there is no other published model that is directly comparable to ours. Our models provide a novel benchmark for future researchers in GDM prediction. We recognise that the performance of our models may require further improvement for use in a clinical setting, for example, by using some intelligent optimization algorithms^40–44^.

To further improve clinical usability, we used feature selection to keep the amount of data entry on the CDSS to a minimum, so that our CDSS would not be time-consuming for clinicians who have heavy workload to fit into their workflow. Our models included a very small set of features (4 or 5) compared with published models (4-2355), which means our models would be easier and faster to use. Moreover, our feature selection process was conducted in collaboration with clinical experts. In our dataset, education level is higher in GDM participants than non-GDM participants, and we trained models on such data which also reflected that a higher education increased the risk of GDM. In literature, conflict evidence was reported on the association of education and GDM. In order to avoid confusion and for our models to translate into clinical practice, education was removed in consultant with clinical experts.

**Table 7:** Comparison with previously published machine learning-based GDM prediction models.

Model	Population	No. of features	Clinical test needed	Specificity	Sensitivity	AUC-ROC	AUC-PR
Model 1	Overweight & obese	5	Fasting blood test (fasting glucose, insulin, white cell count)	0.768	0.733	0.792	0.485
Model 2	Overweight& obese	5	Routine blood test (white cell count)	0.6	0.6	0.659	0.208
Model 3	Overweight & obese	4	No	0.674	0.533	0.656	0.199
Qiu et al.^17^	General	49	Fasting blood test (fasting plasma glucose, complete blood count, liver function test...)	0.998	0.622	0.847	–
Zheng et al.^18^	General	4	Fasting blood test (fasting plasma glucose, triglycerides)	0.75	0.66	0.766	–
Ye et al.^19^	General	17	Fasting blood test (fasting glucose, HbA1c, triglycerides...)	0.99	0.15	0.74	–
				0.26	0.90		
Artzi et al.^20^	General	2,355	Laboratory tests including fasting blood test (glucose, white cell count...), blood pressure measurement, urine test, and blood test in previous pregnancy (glucose tolerance test...).	–	–	0.854	0.318
		9	Blood test in previous pregnancy (HbA1c% test, glucose challenge test/oral glucose tolerance test)	–	–	0.799	0.241
Xiong et al.^21^	General	43	Routine blood test, hepatic and renal function examination, coagulation function examination	0.995	0.883	0.942	–
Yan et al.^22^	General	61	Blood test (complete blood test, liver function test...), urine test (urine glucose, urinary gallbladder, nitrite...)	–	0.706	0.779	–
Hou et al.^23^	General	83	Single nucleotide polymorphism genes, blood test (cholesterol, white blood cell...)	–	–	0.852	–
Wu et al.^24^	General	73	Fasting blood test (fasting plasma glucose, complete blood count, liver function test)	0.82	0.63	0.80	–
		7	Fasting blood test (fasting plasma glucose, HbA1c, triglycerides)	0.82	0.59	0.77	–
Wu et al.^25^	General	15	Blood test (complete blood count, liver function test, ferritin...)	0.269	0.91	0.746	–
				0.524	0.75		
				0.777	0.487		

This is also the first machine learning study to investigate the potential ethnic/cultural difference in GDM prediction. In previous works, all or the majority of the participants are from one ethnic group. Artzi et al.^20^ utilized the electronic health records data in Israel. Hou et al.^23^ trained a model based on data from an unknown population. All others used data collected in a single centre in different cities in China (Sichuan^17,21^, Shanghai^18,19,24,25^, Beijing^22^) and the participants are all or mainly Chinese. For this reason, these researchers did not consider the impact of culture/ethnicity on GDM prediction, and their models may not be applicable to other populations. Our data was collected in a single centre in Dublin, Ireland. The majority of the participants are white (90.70%), but black, Chinese, other Asian and mixed-ethnic women are also included. For the purpose of a novel investigation of the cross-cultural/ethnic difference in GDM prediction in women with overweight and obesity, we trained models on a white population and tested them on non-white women. Our results show that AUC-PR and AUC-ROC are similar between both groups, however, the decision threshold of 0.5 leads to lower sensitivity and higher specificity in non-white women than white women. It shows that a lower decision threshold is required for a non-white population as opposed to a white population in GDM prediction. This is consistent with medical literature, which specifies that GDM is more prevalent in non-white women than white women^45^.

We considered the explainability important in a CDSS. Despite an open debate in literature on whether or not XAI is necessary or worth the substantial cost, the use of XAI has been reported to make CDSSs more acceptable and trustworthy to users, and it may translate into a greater use of CDSSs^11^. In our research, SHAP was applied to explain overall behavior of our models as well as the specific prediction made for an instance. Our case study showed that SHAP generated reasonable explanations for our models. In addition, the effect of features on GDM risk shown in the case study is consistent with medical knowledge. We found out that a high baseline fasting glucose increased the risk of GDM. This is inline with medical literature which suggests median fasting glucose is higher in GDM participants than others^46^. We also showed that a high maternal weight increased the risk of GDM, consistent with the increased risk of GDM with overweight and obesity indicated in literature^2^. Therefore, we anticipate that the explanations would help clinical users to gain insights into the model behavior and increase the acceptability and trustworthiness of our CDSS, and thus enhance the potential for use in a clinical setting. It also fills in the gap of the overall lack of XAI use in CDSSs.

To protect data privacy mandated by the General Data Protection Regulation (GDPR) in EU and the Health Research Regulations (HRR) in Ireland, we avoided the use of instance-based learning algorithms, such as k-Nearest Neighbours (kNN). KNN models are lazy learners and they involve saving the original training set for computation when new data come in. They are suboptimal for a CDSS because they may lead to personal data breaches.

Our work has been implemented into a web server that is currently available for academic use. It resolves the problem of the lack of an open-access model or system for academic benchmarking. Further research is required before adoption into clinical practice. Also, further cross-cultural/ethnic testing, preferably on a much larger sample size, is needed to investigate the optimal decision threshold for different minority cultural or ethnic groups. In addition, future work will be conducted to validate our prototype CDSS in a clinical setting.

## Conclusion

We developed an explainable machine learning-based CDSS for the prediction of GDM in women with overweight and obesity to identify high-risk women for targeted intervention early in pregnancy. The CDSS includes multiple models for theoretical, routine antenatal and remote settings to enhance clinical usability. It also provides explanations for both the structure of the models and each specific prediction to gain trust from clinicians. A web-based prototype of the CDSS is implemented and publicly available for academic use. We also investigated the cross-cultural/ethnic difference in GDM prediction, which implies that a non-white population requires a lower decision threshold than a white population. Further research is required to validate the CDSS in a clinical setting.
